# The Impact of Facilitators’ Competencies and Characteristics on Faculty Enhancement Activities in Saudi Arabia: A Mixed-Methods Research

**DOI:** 10.7759/cureus.54650

**Published:** 2024-02-21

**Authors:** Abdulaziz I Alhassan, Njoud A Alghofaily

**Affiliations:** 1 Department of Medical Education, College of Medicine, King Saud bin Abdulaziz University for Health Sciences, Riyadh, SAU; 2 Department of Research, King Abdullah International Medical Research Center, Riyadh, SAU

**Keywords:** faculty development, training of trainer, training of the facilitators, workshop facilitators, mixed methods study

## Abstract

Background and objective

Seminar-based workshops envision an active learning environment that creates opportunities for faculty to learn how to become effective workshop facilitators themselves. In this study, we employed a mixed-methods design focusing on an in-depth analysis of the data to examine the impact of the competencies and characteristics of a workshop facilitator on faculty development activities and programs.

Methodology

This study involved 159 faculty members and was conducted via a web-based survey and 13 in-depth interviews.

Results

The Pearson correlation coefficients between the three effectiveness ratings showed that all three correlations are significant at the 0.01 level, which signifies a statistically significant relationship between the three variables. The strongest relationship is between facilitator knowledge and facilitator communication, followed by that between facilitator communication and facilitator attitude, and between facilitator knowledge and facilitator attitude. This suggests that the three effectiveness ratings are positively correlated. Qualitatively, four themes emerged in our study: attributes of a good facilitator, participant engagement's role, feedback's impact, and workshop organization challenges. Knowledge, communication, and attitude were identified as effective facilitator characteristics, with knowledge being the most crucial.

Conclusions

Our findings highlight the importance of enhancing participant understanding, providing timely feedback, and bridging theory to practice. Ultimately, effective facilitators strike a balance in terms of knowledge, communication, and attitude, acknowledging the importance of participant engagement and overcoming workshop challenges. We believe our findings will help inform the revision of the current faculty development programs and the development of such endeavors in the future at institutions of higher education.

## Introduction

Teaching in seminar-based workshops is increasingly considered more effective for the delivery of teaching objectives compared to traditional teaching methods [1]. Providing regular faculty development sessions helps foster a model of an active learning environment and creates opportunities for faculty to learn how to become effective workshop facilitators [2]. This can be fulfilled by exposing them to regular training programs comprising workshops and refresher courses, especially for non-professional instructors and beginners [3]. The preeminent actor in integrating technology into education is the teacher, who must orchestrate and supervise a variety of learning processes in the classroom by methodically using technology. Studies have proposed a model using technological tools to enhance faculty learning to accomplish faculty development [4]. The use of technology in faculty development has particularly shown a positive impact in medical and clinical sciences, creating an environment of highly accessible and reliable systems of learning [5-7]. The fact that instructors require specialized competencies and faculty development programs to properly integrate new methods of teaching with digital resources has been recognized [8].

A study has shown that after participating in the Potentially Exploitable Pedagogical Activities (PEPA) program, “in-class activities that use normal pedagogical practices for unpacking and understanding puzzles”, the two English as a foreign language (EFL) teachers showed improvement across all five indicators, which are task orientation, self-efficacy, professional commitment, job motivation, and future perspective, implying that PEPAs have a constructive and positive role to play in developing EFL teachers' professional development. Using practice-as-research as a guide, instructors may utilize PEPAs to build their curriculum [9]. For example, there are three distinct stages in medical education: undergraduate, postgraduate, and continuous professional development for clinicians. Ensuring that society has well-trained, competent, and informed healthcare personnel is critical and is one of the many goals of medical education. The success of this objective is dependent on the continued education of healthcare professionals and academics alike [10].

Several cultural, organizational, and educational factors determine educational attainment among nurses [11]. Basic determinants of shortcomings in nursing education include teachers' lack of familiarity with nursing education, a scarcity of qualified instructors, high salaries for experienced instructors, low salaries for instructors at hospitals, a scarcity of teaching expertise among instructors in general, a lack of variety within educational program outlines, and inappropriate teaching methods [12]. Exhaustion of teaching facilitators due to burnout caused by their involvement in several duties, including teaching as well as working in the ward, inapplicability of certain courses, a mismatch between educational material and class duration, improper class timings, lengthy time spent in schooling, and a lack of group educational opportunities [13]. Another study identified various other challenges to nursing education, such as the shortage of experienced clinical educators, the lack of a thriving learning environment, and the participation of nurses in clinical education [14].

Professional education and training programs for teachers have been developed and implemented in Saudi Arabia in accordance with the Kingdom's Vision 2030 initiative, which includes establishing professional standards for teachers via the National Authority for Education Evaluation. For instance, recent research has contributed to the ongoing effort to learn about the views of English language instructors in Saudi Arabia regarding their existing preparation programs so that they may be compared to worldwide benchmarks for quality [15]. As the implementation of these faculty development programs can be challenging and these programs are often complex, comprising team members from diverse backgrounds, measuring their effectiveness [16], by following up with the participants [17], is of paramount importance. Several studies have been conducted on the efficiency and significance of faculty development programs, both internationally and locally. However, our study, carried out at King Saud bin Abdulaziz University for Health Sciences (KSAU-HS), Saudi Arabia, is one of the first of its kind to investigate how the competencies and characteristics of facilitators affect faculty development activities and programs.

## Materials and methods

Study design

The study involved a cross-sectional web-based survey and qualitative approach comprising in-depth interviews with the study participants. Firstly, an online survey questionnaire was sent to the study participants on WhatsApp Messenger. A total of 159 participants answered the online questionnaire. All incomplete responses were excluded from the analysis.

Study setting and population

KSAU-HS was established in 2005 as the first state university in Saudi Arabia to specialize in medicine and health sciences. KSAU-HS has 14 colleges on three campuses in Riyadh, Jeddah, and Al-Ahsa. The university's mission is to provide excellent health sciences education, health-related research, and community health services. The study was conducted at KSAU-HS, and all the faculty members, clinical and non-clinical, who regularly delivered workshops under the university’s faculty development program or had teaching responsibilities on any of the three university campuses were deemed eligible for participation. The combined strength of the three campuses was 1608 during the study period, of which 420 were non-Saudi and 1188 Saudi Arabian nationals; about 982 were males and 626 were females.

Sample size and sampling methods

For the quantitative part, the sample size was calculated using Raosoft, which amounted to 311 participants, with an assumed level of confidence of 95%, a margin of error of 5%, and a prevalence of 50%. For the qualitative part, the researcher identified ~15 faculty members who regularly conducted the workshops. The final sample size was determined by data saturation. For the quantitative part, a non-probability sampling technique (convenience sampling) was used. For the qualitative part, the non-probability sampling technique (purposive sampling) was employed. The researcher selected the sample based on a pre-determined agenda.

Quantitative data collection

The questionnaire underwent rigorous validation processes, including face validation, construct validation, and content validation. Face validation was conducted through expert reviews and feedback. Construct validation involved testing the questionnaire with a pilot sample to assess its ability to measure the intended constructs. Content validation was ensured through a thorough examination of the questionnaire items by experts in the field. The finalized items were selected based on consensus among the research team and the credibility of members involved in finalizing the questionnaire was established through their expertise and experience in the subject matter.

The questionnaire was tested among a pilot sample of 50 participants, and then its reliability and validity were calculated. The overall questionnaire was proven to be reliable according to Coronach's alpha (Coronach's alpha = 0.866). The Cronbach's alpha of the first section (the effectiveness of the facilitator knowledge) was (0.733), the Cronbach's alpha of the second section (the effectiveness of the facilitator communication) was (0.754), the Cronbach's alpha of the third section (the effectiveness of the facilitator organization) was (0.542), and the Cronbach's alpha of the fourth section (the effectiveness of the facilitator attitude) was (0.716). Hence, we deleted the third section with all its items owing to its weak reliability.

The questionnaire used in this study was self-administered in English and consisted of three sections: knowledge of the facilitator, communication, and attitude. The items were assessed using a 6-point Likert scale, where 0 = not important, 1 = slightly important, 2 = important, 3 = fairly important, 4 = very important, and 5 = extremely important. The participants were sent the link to the questionnaire via WhatsApp.

Reliability test

As mentioned above, we used Cronbach’s alpha test to measure the reliability of our questionnaire. The calculated Cronbach's alpha was 0.922 for all items in the questionnaire and ranged from 0.806 to 0.855 for dimensions. These results indicate a high reliability for the questionnaire because Cronbach's alpha from r = 0 to 1, with r ≥0.7 is considered as sufficiently reliable (Nunnally & Bernstein, 1994) (Table 1).

**Table 1:** Results of the reliability test of the questionnaire

Dimensions	No. of items	Cronbach’s alpha
The effectiveness of facilitator knowledge	5	0.806
The effectiveness of facilitator communication	5	0.827
The effectiveness of facilitator attitude	6	0.855
Instructor/facilitator evaluation	16	0.922

Qualitative data collection

The interviews were conducted individually by experienced data collectors. A total of 15 face-to-face, semi-structured qualitative interviews were conducted using an interview guide in English. Thanks to the pilot study, we were able to fine-tune the interview questions. The questions were open-ended, allowing participants to share their thoughts and experiences in more detail regarding the effect of facilitators' characteristics on faculty enhancement activities in Saudi Arabia. For example, participants were asked structured questions such as "Could you provide a brief overview of your career background?" and "How many workshops have you conducted?" Additionally, questions included: "Did you ensure effective communication by asking questions?" and 'What strategies did you employ to encourage participation from all audiences?" Other questions focused on the importance of paying attention to body language and voice tone; strategies for handling disruptive audiences; challenges faced before, during, and after workshops; feedback mechanisms utilized post-workshop; experiences with co-facilitation; criteria for selecting co-facilitators; characteristics of an ideal facilitator; and recommendations for enhancing facilitator performance. Interviews were audio recorded with the permission of all participants.

Quantitative data analysis

The studied variables included demographic characteristics, and questions related to the characteristics of instructors, The main outcome variables were the instructor characteristic score, which constituted numerical variables that were analyzed as a mean and standard variation based on the total score of 27 questions, while the grouping variables constituted categorical data such as demographic data, which were analyzed as frequencies and percentages. Quantitative data were analyzed in three steps: preparing the data for analysis, exploring the data, and, finally, analyzing the data. Descriptive statistics were calculated to describe the basic characteristics of the study participants and presented as frequencies, percentages, minimum and maximum values, and mean (SD). Inferential statistics were calculated to test the relationship between variables, such as Pearson correlation to estimate the correlation between variables. A p-value of <0.05 was considered statistically significant. We used SPSS Statistics version 29 (IBM Corp., Armonk, NY) for data analysis.

Qualitative data analysis

Taguette qualitative data analysis computer program was used to import the interviews for analysis. For validity, all recordings were transcribed and evaluated by two researchers who independently detected any discrepancies in coding and verified the final themes via discussion. The interviews were transcribed verbatim by the co-author (NG), who then double-checked the interview content with the author (AA). Using the manual transcription method, all interviews were transcribed privately and stored safely. The meaning of the participant expressions in phrases and lines was summarized by assigning the most pertinent and appropriate codes after reading the interview text several times to become familiar with the substance of the data. The rational editing of code names came next. The codes that explained a frequent pattern in the data were then arranged into potential themes. The finalized coded text was exported into a Word document together with the coded data, code names, and participant references. The codes were merged and collapsed into appropriate themes. Before interpretation and editing were done to describe the results of the analysis, the text was read again for any potential errors in coding or topic assignment (theme review). Finally, the theme interpretation and further analysis were performed. Moreover, to add credibility to interpretations of data, the analyses were shared with participants to confirm the accuracy and truthfulness of these interpretations.

## Results

Quantitative findings

Demographic Characteristics

Table 2 shows the demographics of the 159 participants in the instructor/facilitator evaluation. Most of the participants were female (n=95, 59.7%), while 64 participants (40.3%) were male. Regarding years of experience, 25 participants (15.7%) had one to five years of experience, 76 participants (47.8%) had 5 to 10 years of experience, and 58 participants (36.5%) had more than 10 years of experience (Table 2).

**Table 2:** Demographic characteristics of participants in instructor/facilitator evaluation (n=159)

Demographics	Groups	N	%
Gender	Male	64	40.3
	Female	95	59.7
Experience	1-5 years	25	15.7
	5-10 years	76	47.8
	More than 10 years	58	36.5

The Effectiveness of Facilitator Knowledge

As shown in Table 3, the facilitator was rated highest for their ability to have a "specific, mergeable, reliable, realistic, and time of learning objective workshop" (mean = 4.19). They were also rated highly for their ability to cover all the aspects of knowledge related to the workshop title (mean = 3.90). The facilitator had room for improvement in their ability to link the theory to practical action (mean = 3.72). Overall, the facilitator was rated very well, with a mean effectiveness of 3.88. The facilitator could improve their ranking by linking the theory to practical actions. The facilitator could improve their ranking in this area by providing more examples and exercises that help participants apply the theory to their work. The facilitator should link the prior knowledge of the attendees with the knowledge that will be delivered in the workshop. The facilitator could improve their ranking in this area by asking participants questions about their prior knowledge and then using that knowledge to build on what they are learning in the workshop (Table 3).

**Table 3:** The effectiveness of facilitator knowledge

	N	Minimum	Maximum	Mean	Std. deviation	Rank
Q1: The facilitator should be an expert in the field of the workshop content	159	1	5	3.84	.945	3
Q2: The facilitators should cover all the aspects of knowledge related to the workshop title	159	1	5	3.90	.943	2
Q3: The facilitator should link the theory to practical action	159	0	5	3.72	1.055	5
Q4: The facilitator should link the prior knowledge of the attendees with the knowledge that will be delivered in the workshop	159	1	5	3.75	1.085	4
Q5: The facilitator should have specific, mergeable, reliable, realistic, and time of learning objective workshop	159	0	5	4.19	1.137	1
The overall effectiveness of facilitator knowledge	159	1.00	5.00	3.88	.777	---

The Effectiveness of Facilitator Communication

As displayed in Table 4, the facilitator was rated highest for their ability to make the communication enthusiastic (mean = 4.31). They were also rated highly for their ability to show clear body language (mean = 4.21), know how to convey the message clearly, and for effective voice delivery (mean = 4.16). The facilitator could improve on their ability to tell the audience that they did a good job when they performed a new skill or learned something new (mean = 3.79). Overall, the facilitator was rated very well, with a mean effectiveness of 4.08. The facilitator could improve their ranking by providing positive feedback to the participants when they performed a new skill or learned something new. The facilitators could improve their ranking in this area by being more positive and encouraging to the participants. They could also make a point of acknowledging the participants' achievements, both big and small. By following these suggestions, the facilitator can improve their ranking in these areas and provide a more effective learning experience for the participants (Table 4).

**Table 4:** The effectiveness of facilitator communication

	N	Minimum	Maximum	Mean	Std. deviation	Rank
Q6: The facilitator should show a clear body language	159	1	5	4.21	.970	2
Q7: The facilitator should know how to convey the message clearly, effective voice delivery	159	0	5	4.16	.958	3
Q8: The facilitator should make the communication enthusiastic	159	0	5	4.31	.962	1
Q9: The facilitator should tell the audience that they did a good job when they performed a new skill or learned something new	159	0	5	3.79	1.097	5
Q10: The facilitator should have the ability to minimize distractions during the workshop	159	1	5	3.93	.982	4
The overall effectiveness of facilitator communication	159	.60	5.00	4.08	.765	---

The Effectiveness of Facilitator Attitude

As presented in Table 5, the facilitators were rated highest for their ability to explain the purpose of the exercises (mean = 4.02) and use real-life examples to transfer knowledge (mean = 4.01). They were also rated highly for their ability to encourage the sharing of differing opinions (mean = 4.00). The facilitator could improve their ability to make sure the participant understood the material (mean = 3.93) and give feedback promptly to the audience (mean = 3.77). Overall, the facilitator was rated very well, with a mean effectiveness of 3.92. The facilitator could improve their ranking by ensuring that the participants understood the content. The facilitator could improve their ranking in this area by asking participants questions to check their understanding and providing additional explanations as needed. The facilitator should give timely feedback to the audience. The facilitator could also improve their ranking in this area by providing feedback to participants as soon as possible after they have completed an exercise or task (Table 5).

**Table 5:** The effectiveness of facilitator attitude

	N	Minimum	Maximum	Mean	Std. deviation	Rank
Q11: The facilitator should explain the purpose of the exercises	159	0	5	4.02	.914	1
Q12: The facilitator should make sure the participant understands the material	159	1	5	3.93	.942	4
Q13: The facilitator should encourage the sharing of differing opinions	159	0	5	4.00	.994	3
Q14: The facilitator should use real-life examples to get ideas across	159	0	5	4.01	.958	2
Q15: The facilitator should give feedback promptly to the audience	159	0	5	3.77	1.176	6
Q16: The facilitator should ensure that all audiences can share their opinions, ask questions, and practice	159	1	5	3.81	1.259	5
The overall effectiveness of facilitator attitude	159	1.17	5.00	3.92	.799	---

Results of the Correlation

Table 6 shows the Pearson correlation coefficients between the three effectiveness rating items: facilitator knowledge, facilitator communication, and facilitator attitude. A Pearson correlation coefficient constitutes a measure of the linear relationship between two variables. A correlation coefficient of 1 indicates a perfect positive relationship, a correlation coefficient of -1 indicates a perfect negative relationship, and a correlation coefficient of 0 indicates no relationship [18]. We found a statistically significant correlation between the three variables (p<0.05). The strongest correlation was observed between facilitator knowledge and facilitator communication (correlation coefficient = 0.777), followed by facilitator communication and facilitator attitude (correlation coefficient = 0.666), and facilitator knowledge and facilitator attitude (correlation coefficient = 0.658). This suggests that the three effectiveness rating items were positively correlated, meaning that they tend to occur simultaneously. In other words, facilitators who were rated highly for their knowledge were also likely to be rated highly for their communication and attitude, and vice versa. This means that facilitators who want to improve their overall effectiveness should focus on improving in all three areas.

**Table 6:** Correlation findings between the three effectiveness rating items *Correlation is significant at the 0.01 level (two-tailed)

		The effectiveness of facilitator knowledge	The effectiveness of facilitator communication	The effectiveness of facilitator attitude
The effectiveness of facilitator knowledge	Pearson correlation	1	.777^*^	.658^*^
	Sig. (two-tailed)		<0.001	<0.001
	N	159	159	159
The effectiveness of facilitator communication	Pearson correlation	.777^*^	1	.666^*^
	Sig. (two-tailed)	<0.001		<0.001
	N	159	159	159
The effectiveness of facilitator attitude	Pearson correlation	.658^**^	.666^**^	1
	Sig. (two-tailed)	<0.001	<0.001	
	N	159	159	159

Qualitative findings

**Figure 1:**
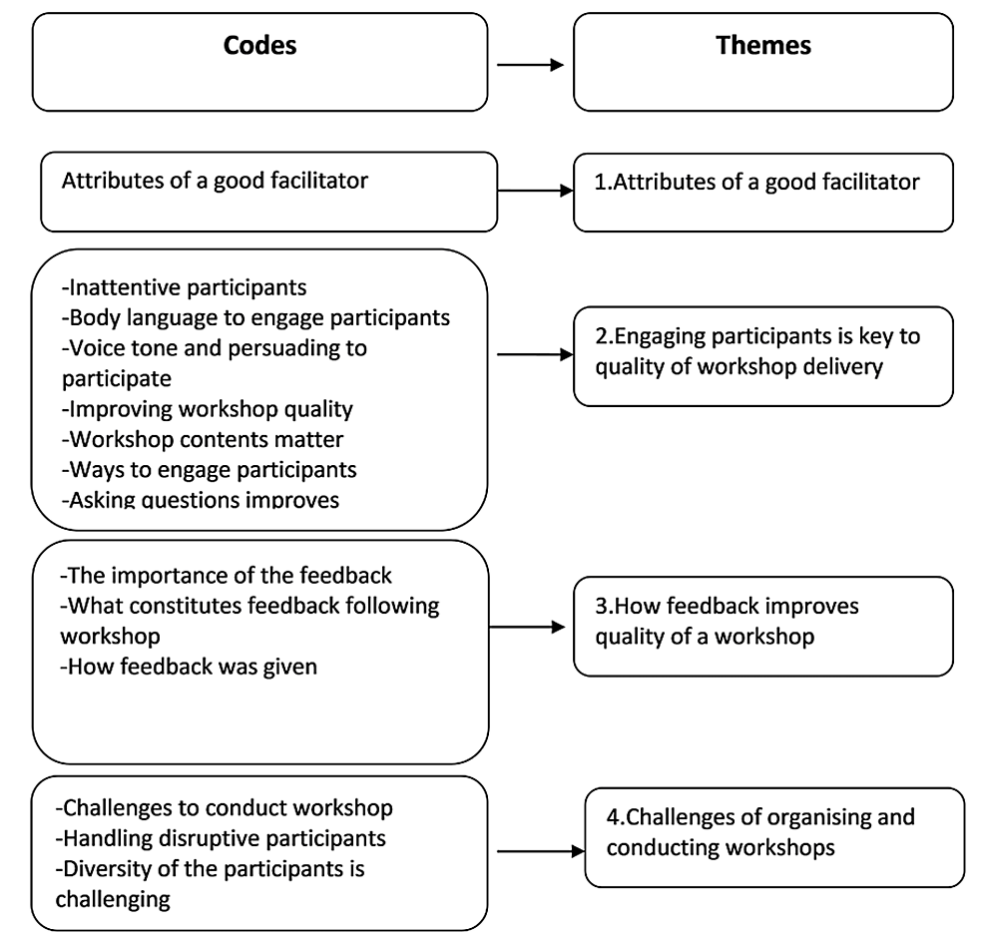
Schematic depicting theme generation

Four distinct but interrelated themes emerged in our analysis. These include (1) attributes of a good facilitator; (2) engaging participants is key to the quality of workshop delivery; (3) how feedback improves the quality of a workshop; and (4) challenges of organizing and conducting workshops (Figure 1). The first theme was based on a single code of a similar name as it consisted of a significant amount of data across all our participant transcripts, and it emerged as a distinct and dominant theme in our data. Seven of our 13 participants were females, and most participants belonged to clinical specialties and facilitated a workshop every month (Table 7).

**Table 7:** Characteristics of the study participants and frequency of conducting workshops

Participant number and profession	Gender	Frequency of conducting workshops
1. Oncology nursing specialist	Male	One workshop a month
2. Nephrologist	Male	Two workshops a month
3. Biostatistician	Female	No count available
4. Clinical dietician	Female	One to two workshops a year
5. Rheumatologist	Female	One workshop every fortnightly
6. Clinical nutritionist	Male	One workshop a month
7. Nurse surgery	Male	One workshop a month
8. Nurse gastroenterology	Male	One workshop a month
9. Nurse orthopedics	Female	Three workshops a month
10. Nurse physical medicine	Female	One workshop a month
11. Nurse neurology	Male	One workshop a week
12. Nurse emergency department	Female	Two workshops a month
13. Nurse immunology	Male	One workshop a month

1. Attributes of a Good Facilitator

In response to our open-ended questions about what the best attributes of the facilitators who conduct academic workshops could be, the participants talked about several qualities that should be present in a facilitator. Many participants believed that a facilitator must use creative methods to involve everyone in the group and encourage group work.

"As a facilitator, your goal is to keep a group on track while not expecting a certain conclusion" (1. Male oncology nursing specialist).

When asked what criteria the participants use to select a co-facilitator in the workshop, one participant said that he would select a facilitator who encourages an inclusive atmosphere in which all participants have equal participation in the group. One participant believed that an ideal and competent facilitator ensures a comfortable environment that encourages the involvement of participants with diverse backgrounds and specialties.

"A competent facilitator must be able to think quickly on his feet. This may imply altering the course in the middle of the process, employing other innovative techniques to involve the group, or soliciting ideas from the participants on how to move the agenda" (10. Female nurse physical medicine).

The establishment of trust and respect between workshop attendees and facilitators was one of the key qualities identified by our study participants. They believed that this necessitated an understanding of how others reacted to both the topics discussed in the workshop and how to respond to the comments and ideas of other attendants in the group. For this to be achieved, they believed that the facilitator needed to have a clear understanding of the types of attendants in the group.

"One method the facilitator learns to detect individuals' sentiments is by attentive listening, both to the stated meaning of words and to their tone as well as implicit meaning (10. Female nurse physical medicine).

"A successful facilitator knows how to deal with each participant and knows how to manage differences between participants professionally" (11. Male nurse neurology).

Awareness of the agenda and time during the workshop was another critical quality listed by our study participants; they believed that the best facilitator is one who is well-informed, attentive to the time allocated for the workshop, and follows the schedule. A participant, a male nurse in immunology, believed that to achieve a high-quality, focused workshop, the facilitator needed to have the ability to be committed to the planned time for the workshops, the ability to deal with the dominant audience and disruptions caused during the workshops, and the ability to improve the focus of the audience on the workshop content.

2. Engaging Participants is Key to the Quality of Workshop Delivery

Participants in our study described situations where their workshop attendees were distracted, and losing focus during the workshop. They cited examples where attendees were not fully attentive during the sessions. One of the study participants, a male oncology nursing specialist, believed that since most attendants of the workshop were clinicians who were preoccupied with their patients, they failed to be fully attentive during the sessions.

Others described how the coronavirus disease 2019 (COVID-19) pandemic affected their attention and presence during the workshop sessions. They believed that since the workshops were delivered remotely, it was difficult to ascertain whether the participants were available or not for the sessions. The participants also believed that sometimes technology and innovative ideas in the presentations could be used to engage workshop attendees who are reluctant to raise their hands and ask questions.

"Not all the time [they were attentive] as, honestly, many of the workshops were [delivered] virtually, most of them were observing social distancing and quarantine during the COVID-19" (4. Female clinical dietician).

The study participants believed that body language was key to engaging the workshop attendees. A participant stated that while good communication skills are essential for success in interpersonal as well as professional interactions, it was nonverbal clues, or "body language," that spoke most loudly. A participant said that body language consisted of gestures, posture, voice tone, and eye contact. These cues send significant messages to the participants. They may either make people comfortable, or they may even be counterproductive and lead to feelings of offense and confusion, leading to poorly conveyed messages during the sessions.

"That the body language must be comfortable for the person you deal with, that you don't seem irritated, and that you are interested in the discussed topic and the given workshop" (4. Female clinical dietician).

Participants talked about how important it was to maintain consistent communication through eye contact and body language with the attendees of the workshop. They believed that many facilitators get caught up in the flow of a presentation and lose eye contact during the sessions. This could be potentially avoided by maintaining constant eye contact and communication. The participants believed that body language was essential to the appropriate delivery of the message of the session.

"You need someone who believes your message and takes action. You must have the right words and a confident tone to get your message across. But if you are fidgeting, look nervous, and don't make eye contact, people may not think you are confident. So, they don't trust your message" (5. Female rheumatologist).

A study participant, a female nurse in orthopedics, believed that body language conveys trust in the audience. It demonstrates a commitment to the task. And the significance of body language lies in how it reflects the personalities of the facilitators. Another participant believed that body language was important for making a connection with the study participant.

"A good body language quickly connects you to your audience or colleagues. No matter how brilliant the subject of the speech is, when you deliver it without emotion or authority, the audience will become bored" (10. Female nurse physical medicine).

The study participants cited several examples of positive body language. Some identified that smiling and establishing eye contact, for example, were simple yet powerful methods to demonstrate that the facilitator was attentive or friendly. These gestures also assist in reducing tension and anxiety.

"Body language conveys a lot about you, each move has a meaning, even when you touch your chin, that also has a meaning. When you can use your body language professionally you can establish strong relations with your audience" (12. Female nurse ER).

Apart from body language, voice tone was another important aspect of communication described by our study participants. A participant, a male nephrologist by profession, described that people read the facilitator’s voice along with paying attention to spoken words; they focus on timing and tempo, how loud the talk is, tone and intonation, and noises that suggest comprehension, such as "ahh" and "uh-huh." The participant specified that the voice tone might convey sarcasm, wrath, tenderness, or confidence, so it must be used cautiously.

Participants described how significant the voice tone was in delivering the message during workshops. One of the participants was particular about specifying when to raise the voice and when to speak in a low voice and selecting the language, English or Arabic, to full effect.

"Also, the voice tone is so important in maintaining the attention of the audience and not to lose it. The tone by which you speak and when to change it, whether lower or higher, is important to draw the attention of the audience" (12. Female nurse ER).

A participant, a male nurse, specified that variation in voice can be used to demonstrate that a facilitator is attentive, sensitive, and considerate. He described that a higher pitch or volume, for example, can be used to express surprise, enthusiasm, or agreement, whereas a lower pitch or volume can be used to express curiosity, concern, or sympathy; a faster pace can be used to express urgency, enthusiasm, or interest; a slower pace can be used to express calmness, importance, or respect; and a warmer or softer tone can be used to express rapport, appreciation, or empathy.

Another participant believed that the size of the group matters if the facilitator can engage the participants. She believed that if the workshop is conventional for those acquiring knowledge, then the number of participants is not limited; however, the number must not exceed nearly 50 participants in one workshop.

"I mean for example, if the number of participants is small, you will give more attention, while if the number of participants is large, you may not be able to give much attention to the participants because of their numbers" (3. Female biostatistician).

One of our study participants, a male nurse in gastroenterology, responded that an engaging workshop session would include some engaging introductory material. For instance, if the facilitator has some research study findings, it will be engaging if he uses the research to have the audience guess some of the findings. Or, starting the sessions with a quiz can help generate discussion. The facilitator must think of some interesting beginnings, as the opening is the most important part of any workshop.

"Before you begin your material, ask your audience for their most frequently asked questions. Tell a tale or convey a case study, but let the audience decide which one to hear" (8. Male nurse gastroenterology).

Our participants also discussed various factors that they believed were crucial to workshop facilitation. They discussed strategies such as training in communication, presentation, and social skills for the facilitators. One of the facilitators, a male nurse in surgery, described that the facilitator’s performance can be improved by taking feedback from the audience in each workshop, which helps to avoid any misunderstanding, improving the outcomes of each workshop, as well as increasing the satisfaction of the audience.

"The problem is that there is nothing you can do to prevent mistakes or minor problems in your workshop. The idea here is to be ready for any eventuality and to be willing to change on the go" (8. Male nurse gastroenterology).

Our participants believed that questioning skills were a key aspect of verbal communication during the workshop, paving the way for highly engaged participants. Asking questions helps you receive answers, exchange knowledge, and maybe show appreciation for the details provided by the facilitator. Some participants directly identified the students to ask questions.

"If I see somebody, somewhere else with a busy mind, I call him by name and then ask a direct question, a simple question just to get attention" (1. Male oncology nursing specialist).

Our participants strongly believed that facilitators asking questions of the workshop participants was key to encouraging group engagement and developing confidence and communication skills. Similarly, they found taking questions from the participants equally important. The participants said that while they are facilitating, they encourage interaction to the extent that they allow students equal time to contribute and ask questions.

"I would spend 10 minutes at the start of each session answering questions concerning the previous day's principles and reviewing complicated issues" (2. Male nephrologist).

Participants preferred to ask open-ended questions because they believed that it was a better way to learn more about a given issue, as an open question allows others to elaborate and provide a detailed explanation. However, to avoid repeating the same questions over and over, participants said that the facilitators must listen to the responses. If a facilitator inadequately responds to participants' questions, they may become frustrated, which might lead to a loss of attention.

"The first question: what's on your mind? A strong starting question is essential and asking a topic like this will get the conversation started fast and effectively; no requirement for unnecessary chit-chat" (13. Male nurse immunology).

3. How Feedback Improves the Quality of a Workshop

Participants discussed the undeniable role that feedback plays in achieving the workshop objectives. For our participants, the feedback was presumed to be something that reflected on the performance of the workshop attendants, but it was mainly what the attendants thought about the performance of the facilitators themselves.

"When done correctly and with the appropriate objectives, feedback may lead to exceptional performance. The audience must be aware of what they perform well and what they do incorrectly" (1. Male oncology nursing specialist).

Participants talked about what constitutes genuine feedback from the facilitators. They believed that a facilitator must encourage a feedback-friendly environment. The facilitator should ask questions such as "Do you understand?" and "Do you have any doubts?" Feedback is necessary in communication to determine if the recipient comprehends the message of the training. The participants believed that the feedback was critical to learning and growth. It was important to give a clear idea of what a good job a facilitator was performing and what was required to improve in the future.

"In addition, correcting errors or reinforcing responses in the process of learning benefits learning on three levels: autonomy, self-direction, and greater embedding of received knowledge" (4. Female clinical dietician).

Our participants stated that feedback was essential to enable attendees to assess their performance and know what points of weakness they have so that they can improve their knowledge-seeking abilities. They also talked about the negative consequences of an absent feedback system. In its absence, professional growth is delayed, and negative behaviors are frequently neglected and become permanent habits.

"Providing feedback to the audience enables them to become better in the part in which they are interested, feedback is essential for encouraging the audience to improve their skills in communication and dealing with others" (12. Female nurse ER).

Participants in our study also discussed appropriate and effective ways to deliver feedback. A participant said that for the attendees to clearly listen to suggestions for improvement, feedback must be presented thoughtfully and regularly. This participant also believed that imparting feedback is an acquired skill, and like any skill, it takes practice to become an expert in it. Another participant said that she preferred direct feedback as it was most effective.

"I may tell the participants that I hope I benefited them, that they learned from me, and that if any participant performed something in that workshop or program, then they can contact me and share their progress with me later; something like that" (3. Female biostatistician).

Some participants explained that most of the time, this feedback is not structured. For example, a participant said that for nurses, feedback is given verbally but not by using an evaluation form, and it is only a recent trend that feedback has been given using online forms.

"They do an evaluation form, and then they choose, for example, the best speaker from those who gave all the workshops (6. Male clinical nutritionist).

One of the participants, a female nurse in orthopedics, believed that the best method for providing feedback was to show interest in what participants achieved in the workshop, encouraging them, making them feel confident, and allowing them to be more successful, as well as enabling them to be more interested in receiving further training. Other participants even said that they frequently followed up with their attendees about the feedback given.

"After providing feedback to an attendee, I frequently follow up with a self-reflective inquiry, "How did that go?" Which opens the door to more conversation. I give feedback to my participants through their emails on the final day of the workshop" (9. Female nurse orthopedics).

Our participants believed that the ideal method for giving feedback was to communicate with the audience the issues encountered by each of them, but not in a personal-directed manner. It is better to communicate the overall issues and how to avoid them. The best way to describe it was through printed notes sent to each participant, which include a handwritten part that is left empty until after the workshops when it is filled for each participant separately after discussing their opinion of the facilitator’s performance in the workshop. Therefore, while most participants in our study preferred to give direct feedback, some also liked to give feedback indirectly.

"Then, I tell them about their pitfalls but not in a direct way; I tell them each pitfall and why I want them to improve on that point" (10. Female nurse physical medicine).

4. Challenges of Organizing and Conducting Workshops

In this theme, we examined the data about a range of challenges that our participants described as facing during their workshops. Participants described several administrative challenges that they encountered during their workshops. These included staff shortages as some assistance is required during the workshops and funds shortages because several little expenditures unexpectedly occur during the workshop planning process. A participant also described the issue of having more attendees than allocated for the workshop, which he said creates challenges in running workshops smoothly. Participants often categorized these workshop-related challenges into before, during, and after the workshop.

"Setting a timetable for the workshop, the different cultures of the audience, preparations for the topics" (5. Female rheumatologist).

One of the challenges described was time management and the scheduling of the workshops. This was because the participants were not all available at the same time. And, the attendees keep updating their content every month to make it more organized. Similarly, identifying useful content for the workshop was also found to be more challenging.

"Thinking of new information is also one of the big challenges" (6. Male clinical nutritionist).

Before the workshop, therefore, the main challenge was to gather materials, prepare the presentation for the workshop, develop the overall budget needed for the workshop, and identify methods available for registration for the workshop.

"The cost of the materials is too difficult, that you must deal with and maintain the deal with the sources that provide the workshop materials. Also, buying the materials wastes a lot of time" (4. Female clinical dietician).

A participant, a male nurse in Neurology, explained that locating a suitable place for the workshop, preparing a highly impactful PowerPoint presentation, reaching out to the targeted audience, and establishing a timetable for the workshop were some of the challenges that he faced before organizing the workshops.

"Identifying all the materials needed for the workshop, the workshop place and whether it is in a hospital or outside, and the limited number required of attendees [are some of the challenges]" (13. Male nurse immunology).

During the workshop, participants were found to be concerned about their attendees not interacting with them or not being interested in the topic discussed. They said they were worried that they had a bored feeling during the workshops, which may reflect on the audience. Most participants shared this concern. They emphasized that losing the attention of the audience and the presence of too many attendees were concerns for them during the workshops.

"This is a challenge for me that I would like that, after the workshop or the lecture, all the audience are satisfied and happy. That they interact and talk with me in the workshop" (6. Male clinical nutritionist).

Some participants also talked about external distractors during the workshops, which they said negatively affected the workshop. This is because the workshop participants were often distracted by their other duties and responsibilities, leading to a loss of control over the audience and boredom for others.

"Avoiding boredom to have the attention of the attendees throughout the workshop, development of creative ideas to have an interactive audience [are some of the challenges]" (10. Female nurse physical medicine).

After the workshop, the most common concerns were the lack of feedback from the audience, the absence of discussions on the issues encountered during the workshops, and educating the audience on how to remember the discussed information. Some also identified the challenge of providing the workshop participants with relevant workshop material.

"Inability to take feedback from all the audience, finding the most suitable way to provide the attendees with the workshop materials and other possible references, implementation of audience’s recommendations [was challenging]" (7. Male nurse surgery).

Some other participants said that planning for new topics for further workshops, dealing with negative feedback, and meeting with senior facilitators to learn from them for upcoming workshops were challenging.

"Recapping information in a short time to help the audience recollect information. Keeping contact with the audience to help them in answering further questions was challenging" (10. Female nurse physical medicine).

Disruptive audiences were also one of the challenges described in our study. These disruptions included side talks, outside noise, or sometimes disruptions from the participants’ workplaces. Participants also discussed several techniques that they use to prevent disruptions in their sessions. They believed that attendees are less inclined to interrupt a discussion if they believe they helped shape it. It is always better to send out a planned agenda in advance and solicit feedback from my participants about the agenda.

"Although an agenda does not eliminate interruptions, it does serve as the foundation for my intervention" (2. Male nephrologist).

While some of the participants said they usually ignore disruptions in the session, most others took a proactive approach to stopping disruptions. A participant said she would ask a simple question that an attendee could answer, and by the end, he would follow up on that. These sudden questions on the workshop topic make them attentive again. Another participant said the best way to handle disruptive audiences is to ask them why they are talking about topics unrelated to the workshop topic and to also ask them what could help them refocus their attention on the discussed topic. Another participant recommended that it is important to understand the type of audience and personalities attending the sessions.

"Another successful method is removing the source of distraction, for example, outside radio songs or a television that is turned on in a nearby room" (13. Male nurse immunology).

"To deal with a difficult audience, start recognizing signs of resistance" (5. Female rheumatologist).

A minority of our study participants believed that attendance from various backgrounds and professions was a concern for them as it was difficult to conduct a workshop at the same level for all participants simultaneously. The participants belong to different specialties in the hospital.

"Our attendees have different backgrounds, how to understand the taught topics, this is one of the biggest challenges to be honest" (1. Male oncology nursing specialist).

Results of mixed-methods research

To compare the findings from quantitative and qualitative data, the results are combined in this section. Three main areas of findings emerged in the data, which are presented here for comparison (Table 8).

**Table 8:** A Comparison of the findings from quantitative and qualitative components of the data

Three areas measured	Data sets		
	Quantitative result	Qualitative results	Mixed results
Facilitator knowledge and communication	Significant positive correlation between knowledge and communication	Facilitators indicate the positive relationship between knowledge and communication	Similar and convergent
Facilitator knowledge and attitude	Significant positive correlation between knowledge and attitude	Facilitators indicate the positive relationship between knowledge and attitude	Similar and convergent
Facilitator communication and attitude	Significant positive correlation between communication and attitude	Facilitators indicate the positive relationship between communication and attitude	Different and convergent

1. Facilitator Knowledge and Communication

The quantitative analysis showed a high correlation between facilitator knowledge and facilitator communication (correlation coefficient = 0.777, p<0.001), indicating that a higher level of facilitator knowledge was linked with a high quality and level of communication and delivery of the workshop content to the participants by the facilitators. Qualitative analysis also supports this correlation where “engaging participants is key to the quality of workshop delivery” was a dominant theme. Communication was found to be an important determinant of workshop delivery. For example, one participant, a female biostatistician, listed some qualities of an ideal facilitator. For her, the ideal facilitator is the one who understands well what he is doing and saying, speaks with a clear and loud voice, whose speech is organized chronologically, in line with the presented presentation; who prepares creative presentations; has patience; is not too friendly with the participants; communicates effectively; and maintains a high-quality voice tone and body language. Another participant stressed the need for a high level of communication skills for the workshop facilitators.

"He should also have good communication skills and be able to have active interaction with the audience during presentations. A good facilitator is a good listener as well, he must have the ability to listen to the thoughts of different audiences" (5. Female rheumatologist).

Another participant, a female clinical nutritionist, described that the materials that the facilitator presents must be of high quality. She suggested that if the presentation is well prepared and well organized, then the facilitator is truly interested in the topic. She also believed that continuous education about delivering workshops for high-level professionals was important. Participants believed that good communication was dependent on high-quality knowledge of the subject. A participant, a male nephrologist, discussed how feedback by the facilitator to the attendees emphasizes the quality of the learner's work, encourages, and challenges the attendees to improve their knowledge and skills, and identifies what was misunderstood or not understood. They were also interested in giving feedback to the participants that they thought encouraged them to communicate more effectively.

"Effective feedback encourages learners to reflect upon their learning, and learning practices to improve their learning progress, their performance, level of knowledge, their background of the topic, their performance, weaknesses, and strengths" (3. Female biostatistician).

Several of our study participants talked about how important the training of the facilitators was for their knowledge. They discussed how a facilitator’s performance can be improved through refresher training and sustained practice, sharing in different workshops with different facilitators, and using opportunities for continuous online learning. A participant said that a facilitator’s knowledge can be improved when he considers feedback from the audience and listens to their advice and opinions, thereby communicating effectively.

"I think that training sessions by elder facilitators must be given to younger facilitators to enhance their performance; on how they deal with participants, how to have interactive workshops and how to maintain the attention of participants" (11. Male nurse neurology).

2. Facilitator Knowledge and Attitude

Our quantitative findings showed a moderately strong correlation between facilitator knowledge and facilitator attitude (correlation coefficient = 0.658, p<0.001). This shows that high-quality communication was also linked to a high-level knowledge of the facilitator. The facilitator's attitude comprised the ability to explain the purpose of the exercises, use real-life examples to get ideas across, and ability to encourage the sharing of differing opinions. Our qualitative analysis also identified the facilitators’ attitude-related characteristics, which were linked to their knowledge. One of our participants in the study, a female nurse in orthopedics who regularly conducted academic workshops, stated that a good facilitator should have an open attitude toward the use of modern methods of teaching to upgrade the knowledge of participants. Self-improvement and knowledge acquiring were the other qualities that our participants identified as dominant attitudes of any workshop facilitator.

"The successful facilitator should never stop learning, continuous learning enables continuous success, the facilitator should meet with other facilitators from all over the world to allow sharing of their experiences" (9 Female nurse orthopedics).

Many facilitators talked about how attitude impacts knowledge and vice versa. A participant, a female nurse in physical medicine, explained that she often provided the participants with the tools, links, and access that they needed to publish live blog streams, chats, and event spotlights on their own websites or social media, allowing them to promote and drive dialogue around presentations for further discussion. Another participant discussed how to identify an engaged audience in the workshop.

"An engaged audience is one that gains more knowledge and has an overall sense of being appreciated. If they had a wonderful time with you, they are more likely to return for the next workshop. They are more inclined to inform others about what they learned or to share good social media posts about it" (13. Male nurse immunology).

Another participant, a female rheumatologist, described that the workshop facilitator's abilities include a positive attitude toward understanding the mood of the sessions, asking questions in the group, and adapting based on the situation. This was one of the reasons why most facilitators have improved the outcomes of their workshops. Another participant described that there were several ways to accelerate knowledge delivery and evolve to become a professional facilitator. These included finding opportunities to see other presenters' work, learning from a peer group with a few interested people in the same area to help each other learn, moderating with an experienced moderator, discovering new tools and techniques, and receiving training on specific facilitation frameworks and facilitation skills.

3. Facilitator Communication and Attitude

Our quantitative findings showed a moderately strong correlation between facilitator communication and facilitator attitude (correlation coefficient = 0.666, p<0.001). In qualitative analysis, we found that the facilitator must possess a positive attitude toward high-quality teaching and training. They also talked about the ability to set clear objectives for each workshop and professional communication skills that attract participant attention. Participants in this study also discussed the importance of maintaining a positive attitude toward ensuring healthy communication between workshop attendees. A male nurse in gastroenterology suggested that the facilitators need to have the ability to develop and maintain a healthy environment where all participants can effectively communicate. This, he believed, would need a positive attitude for starting discussions, embedding creative teaching methods, and showing some flexibility.

"[He] is not rigid in the teaching process and allows the needed conversation to take place for advancement" (8. Male nurse gastroenterology).

For effective delivery of messages in workshops, some participants talked about the importance of effective sharing of opinions. One participant, a male nephrologist, described that interactive workshops consisted of activities and examples prepared for groups of attendees who worked together to solve complex problems. Therefore, a variety of workshop delivery techniques, such as small-group talks, scenarios, simulations, movies, games, and role plays, can have a significant impact on the delivery of messages. Other participants believed that the topic, not necessarily the content, of the workshop must be attractive.

"It doesn’t matter, the most important thing is the topic, the workshop's topic. In the end, if I'm not interested in the topic, then I would take the information I need" (3. Female biostatistician).

Our study participants identified that activities like summarizing and refocusing, recapping the major points or arguments presented, and reaffirming the purposes can ensure effective communication and improved learning. A male oncology nursing specialist believed that engaging techniques could include posing a challenging question to the participants or just restating the main conclusion at the end of the session. Another participant believed that allowing time to ask questions in the sessions could help improve interaction and participation.

"During interactive discussions, I allow students equal time to contribute and raise questions. Sometimes I deliberately call on students to promote their participation so that everyone can have an equal opportunity to participate in the discussion" (2. Male nephrologist).

## Discussion

This study used the mixed-methods approach to understand how the competencies and characteristics of a trainer or a workshop facilitator affect the effectiveness of faculty development activities and programs at an institute of higher education in Saudi Arabia. Specifically, in the quantitative part, we aimed to identify what set of characteristics of a facilitator drive effective faculty development training, and in the qualitative component, we aimed to explore the competencies and characteristics of the facilitator to provide an effective workshop. This mixed-methods approach helped us understand the aspects of the study that may not have been uncovered by quantitative inquiry alone. The qualitative approach not only complemented our quantitative results but also validated them.

Our study identified traits of the facilitators that were linked with their effectiveness as reflected by their high rating. We found that the facilitators could improve their ranking by being more positive, encouraging, and innovative, and by providing a more effective learning experience for the participants. Our results are in line with a study that also emphasized traits like congenial attitude, being respectful towards the participants, providing critical insight, managing conflicts, and possessing high-level communication skills. This study also showed that creating a congenial learning environment was associated with a more effective delivery of content [19]. Another qualitative study has also shown that a high level of skill and a sense of purpose were linked with higher motivation among facilitators [20].

In our study, the facilitators were rated highest for their ability to conduct workshops based on SMART objectives. Studies have shown that workshops are determined to be successful when the trainees are aware of the predefined and clear objectives of workshops. The trainees have high expectations that the facilitators should identify the appropriate members to be made part of the team and discuss workshop topics. Therefore, efficient facilitators always deliver to the expectations of the trainees [21]. This also leads to the development of critical thinking and the ability to ask questions and improve learning [22].

Our study showed that the facilitators were rated high for their ability to cover all aspects of knowledge related to the workshop topic. One study showed that such a training program for faculty could be effective in improving the knowledge, skills, as well as attitudes of the participants [23]. This knowledge could include expertise in the topics covered, capacity for teaching, awareness of the teaching methods, fair assessment and evaluation, and management of the group in a classroom [19]. Another study recommended that the facilitators must use this knowledge to provide the participants with references and other resources for learning. Therefore, knowing the topic is crucial to being effective in the workshop delivery [21].

Our study identified that the facilitators who were rated highly for their knowledge were also rated high for their communication and attitude. Our results are consistent with a study that showed that the attitude of the facilitator matters in the achievement of learning outcomes. A positive learning environment and availability of essential resources are important for such favorable outcomes of teaching [23]. Clear material and participant engagement were some other related attitudes that contributed to the effectiveness of facilitators in our study. This aligns with a study that showed that facilitators who paid attention to learning environments where participants could communicate effectively and engage within groups were more effective [24]. Engagement and communication of the facilitators with students and across teams are essential to achieve successful teaching outcomes [19]. In another study, the participants benefited from groups where they were provided opportunities to engage with their peers and teachers [1]. Therefore, apart from learned staff, actively engaging and supportive facilitators are key to the success of the workshops [10].

Like engagement, timely feedback was a dominant area highlighted in both quantitative and qualitative data in our study. Our study concurs with other literature on the importance of feedback in the delivery of an effective workshop. Feedback needs to be timely and regularly, optimally given around the time of completing the assignments and evaluations. Timely feedback therefore is important for effective rectification of any deficiencies in the learning process [21]. Another study revealed that for successful learning from these trainings, honest and fair evaluation and feedback by the mentors and facilitators is crucial [17]. Yet another study showed that a focus on the learning process is linked with providing opportunities for reflection, and therefore feedback [24].

Our participants identified some of the challenges of organizing workshops, one of which is the time spent on scheduling and organizing the workshops and finding resources. Studies have shown that facilitators need more time and support to focus on the key tasks of delivering the workshops instead of just organizing them. Other studies have also shown that facilitators elsewhere also spend more time coordinating instead of directly mentoring the participants [24]. The diversity of the participants was another issue highlighted by our participants in the qualitative study. A study has recommended that any faculty development program can be best offered as a structured workplace-based learning matching the needs of the particular type of faculty to avoid any issues because of the diversity of the group [23].

Strengths

This was an innovative study, and the mixed-methods approach is a major strength of this study. No other study from the region has previously investigated the impact of the effectiveness and characteristics of the facilitators on faculty development workshops using by a qualitative or mixed-methods approach.

Limitations

One of the limitations of this study is that caution should be exercised in generalizing the findings because of the qualitative component of the study. Another limitation is that the data for the quantitative component was collected using an online survey, which may not be truly representative of the actual target population. Nevertheless, this study provides basic data on the subject and can help inform the development of further studies in the country and other similar settings.

Practical implications

As our study highlights the fact that the effectiveness of the facilitators is determined by their attributes, engaging participants is key to the quality of workshop delivery; also, feedback improves the quality of a workshop, and the challenges of organizing and conducting workshops need to be overcome to improve the quality of learning in faculty development workshops.

Recommendations

When conducting workshops, it is imperative to prioritize participant engagement as a cornerstone for ensuring quality sessions. Actively involving participants fosters a dynamic learning environment and enhances the overall effectiveness of the workshop. Additionally, emphasizing the importance of feedback is crucial for continuous improvement. Feedback mechanisms enable facilitators to identify areas of strength and those needing improvement, thereby refining workshop content and delivery methods. Moreover, addressing challenges related to organizing and conducting workshops is essential for optimizing effectiveness. By proactively identifying and mitigating obstacles, such as time constraints or resource limitations, facilitators can ensure smoother execution and better outcomes for workshop participants.

## Conclusions

This mixed-methods study aimed to investigate how a workshop facilitator's or trainer's competencies and traits impact the efficacy of programs and activities for faculty development. The facilitators received the highest rating overall for their ability to lead workshops, cover every facet of the subject matter, and improve communication within the sessions. By asking participants questions to gauge their understanding and providing additional explanations as needed, the facilitators could improve their ranking by giving more examples and exercises that help. They could also be more creative, positive, and supportive of the learning process, and they could ensure that the participants understood the material and could link the theory to practical action. We believe this study effectively addresses the research question about the effectiveness and characteristics of the facilitators by identifying key factors that are linked with effective workshop organization for faculty development.

## Figures and Tables

**Figure 2:**
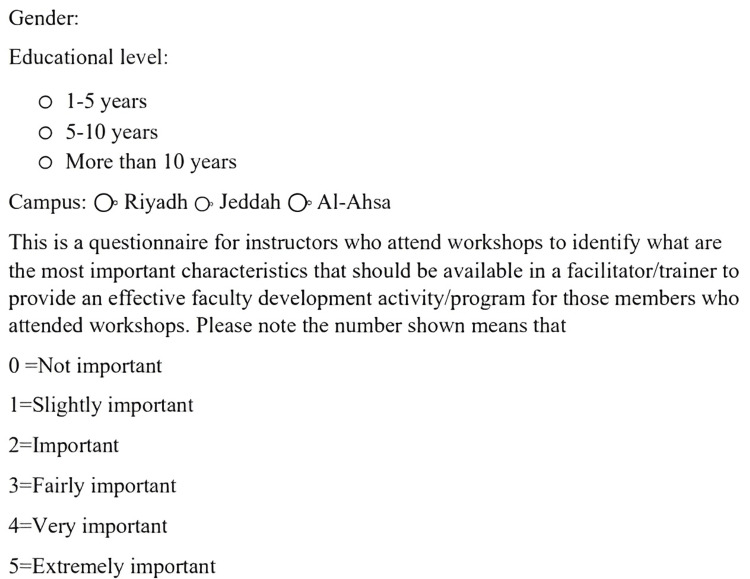
Instructor/facilitator evaluation questionnaire

**Figure 3:**
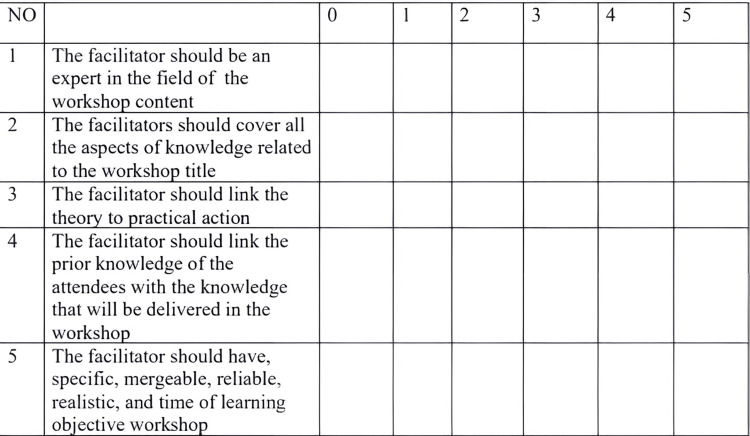
The effectiveness of facilitator knowledge

**Figure 4:**
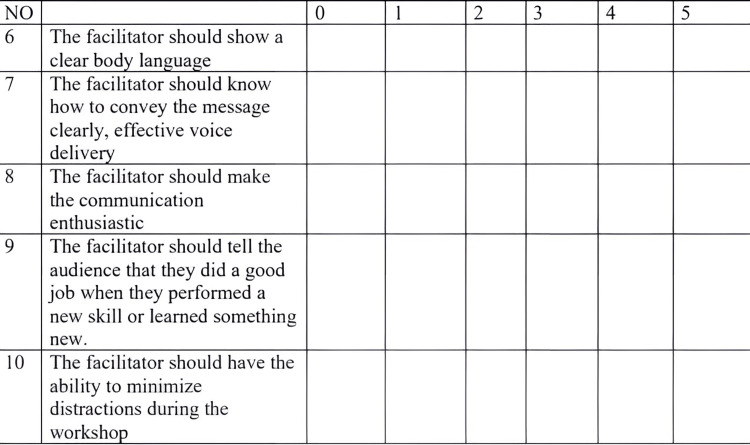
The effectiveness of facilitator communication

**Figure 5:**
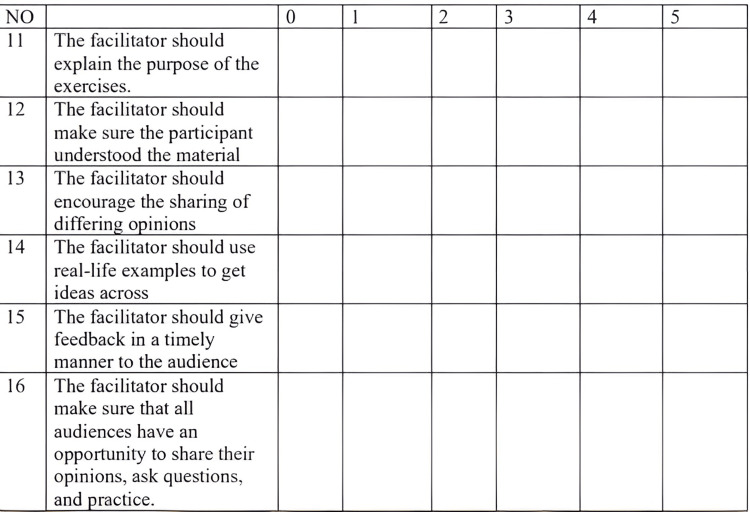
The effectiveness of facilitator attitude

## Appendices

This is an interview guide for the participants in the English language.

1. Describe your career background briefly. How many workshops do you conduct?

2. Did you ask questions to be effective at communication?

3. Did you make sure that all audiences are participating and what was your strategy to encourage them to participate?

4. To what extent the facilitator should pay attention to the body language and the voice tone?

5. What is your strategy to handle disruptive audiences?

6. What challenges did you struggle with as a facilitator before, during, and after the workshop you conducted?

7. Do you give feedback after the workshop? If yes what is the best way of providing feedback to the participant

8. What is the importance of providing effective feedback to the participant?

9. Did you try to conduct a workshop with another facilitator? If yes, what were your criteria for selecting the facilitator to help you with your workshop?

10. What do you think is the characteristic of the best facilitator?

11. What do you recommend to enhance the performance of the facilitator?
